# Identifiability, exchangeability and confounding revisited

**DOI:** 10.1186/1742-5573-6-4

**Published:** 2009-09-04

**Authors:** Sander Greenland, James M Robins

**Affiliations:** 1 Department of Epidemiology and Department of Statistics, University of California, Los Angeles, CA 90095-1772, USA; 2 Departments of Epidemiology and Biostatistics, Harvard School of Public Health, Boston, MA 02115, USA

## Abstract

In 1986 the International Journal of Epidemiology published "Identifiability, Exchangeability and Epidemiological Confounding". We review the article from the perspective of a quarter century after it was first drafted and relate it to subsequent developments on confounding, ignorability, and collapsibility.

## Introduction

Nearly a quarter of a century ago, we published an article titled "Identifiability, exchangeability and epidemiological confounding" [1], hereafter IEEC. At the request of the editor of the present journal, we review the article in light of the extensive developments since that time. In brief, the article gave a formal definition of confounding and a logical justification for correct intuitions about confounding that existed before. Among its deficiencies were that it failed to give adequate historical context and it was not general enough in its discussion. Greater generality was achieved in subsequent articles, especially "Confounding, collapsibility, and causal inference" [2] - which in some ways was an expansion and extension of IEEC addressed to a statistical audience - and in the less technical epidemiologic article, "Estimating causal effects" [3].

Since then, some brief histories of confounding have appeared [4-7]. There have been many conceptual and technical developments as well; below we will cite a few of them that have been the focus of our research in recent decades.

### Confounding Discussions Before IEEC

#### Confounding Before the 1980s

Like nearly all work before the 1980s, IEEC dealt only with estimating effects of exposure histories that could be captured adequately by a single summary, such as "exposed" and "unexposed." IEEC is in some ways no more than a logical finale to a long history of development regarding simple exposures. Concepts of confounding can be traced far back, e.g., John Stuart Mill discussed issues of confounded comparisons in his famed treatise on inductive logic [8]; so did Yule (p. 134) [9] under the heading of "fictitious association." The key concepts were well known to many observational researchers in sociology and epidemiology long before we entered the field (e.g., [10-14]), although not always under the rubric of "confounding" - "spurious association" was a common term for the same idea.

Regardless of terms, confounding is the problem of confusing or mixing of exposure effects with other "extraneous" effects: If at the time of its occurrence, exposure was associated with pre-existing risk for the outcome, its association would reflect at least in part the effect of this baseline association, not the effect of exposure itself. The portion of the association reflecting this baseline association was called confounding or "spurious association." The factors responsible for this confounding (those producing the differences in baseline risk) were called *confounders*.

A few issues were not completely clear to some practitioners. One problem was that many researchers were inclined to overlook the *pre-existing *(baseline) proviso in the description. It was not unusual to see causal intermediates (causes of disease affected by exposure) treated as confounders; unfortunately, this practice adjusts away part of the very effect under study and can induce selection bias even under the null hypothesis of no direct, indirect, or overall effect of exposure. This problem was especially common in cardiovascular research, where studies of diet and lifestyle often adjusted for clinical measurements (blood pressure, serum cholesterol, etc.) taken after the behaviors in question. Apparently, exhortations against adjustment for post-exposure variables in randomized experiments (e.g., [15]) had not effectively filtered from experimental statistics into epidemiology.

To explain the problem, a number of authors (e.g., [14,16-18]) illustrated and contrasted confounders with intermediates using causal diagrams (then known as path diagrams). IEEC used a different mode of illustration, one imported from experimental statistics: The potential-outcomes model.

#### Potential Outcomes

The potential-outcomes model of causation, also known as the response-schedule or "counterfactual" model, was first formalized by Neyman in 1923 [19] (who soon thereafter teamed with Egon Pearson to develop the theories of alpha-level testing and confidence intervals). Of primary interest in IEEC, the model supplied justifications for a number of intuitions about epidemiologic confounding that existed before the 1980s, as well as the nonintuitive ideas that randomization did not guarantee absence of confounding [20], and that confounding did not correspond fully to the statistical notion of noncollapsibility [21]. Thus the model is worth reviewing in detail.

As described in many articles and books (e.g., [22-26]), the model encodes causal statements by assigning to each study unit (whether a person, cohort, or population) a different outcome variable for each exposure level. For a binary exposure indicator X, the familiar "Y" of associational (noncausal) regression analysis is replaced by a pair of variables Y_1_, Y_0 _representing the unit's outcome when exposed and the unit's outcome when not exposed, respectively: Y_1 _is the outcome when X = 1, Y_0 _is the outcome when X = 0. Unit-level effects are differences or ratios of these exposure-specific and unit-specific outcomes. For more general X (including multivariate treatments, wherein X is a vector or even more complex), Y_x _represents the outcome if X = x. As did IEEC, for simplicity we will focus on the binary X case, noting that our comments apply generally.

With this model, the problem of causal inferences devolves to how one can identify these effects when for each unit at most one of the outcomes can be observed. In other words: How can we estimate an effect such as Y_1_-Y_0 _when we cannot observe both Y_1_, Y_0 _at once? Strong assumptions are needed. Randomization was a natural assumption for experimental research, and by the 1930s the potential-outcomes model was established in experimental statistics (e.g., [27]), although without that name. It was sometimes called the "randomization model" [28]; nonetheless, randomization was an assumption for its use, not intrinsic to the model itself, and by the 1970s the model was being used for nonrandomized studies.

Rubin [29,30] introduced the term "potential outcomes" and formalized a set of assumptions that identified average causal effects within the model. An important part of Rubin's formulation was to link the causal-inference problem to the missing-data problem in surveys: Under the model, at least one of the potential outcomes is missing. IEEC however was based instead on the formulation spelled out by Copas (p. 269) [28], which had been common currency in studies of randomization-based statistics before the 1970s (e.g., [27,31]). IEEC never used the term "potential outcome" or cited Rubin's work, which at the time was not familiar (nor apparently to the reviewers). This lapse was rectified in later updates (e.g., [2-4,23]).

The potential-outcomes model has often caused consternation because it treats the potential outcomes Y_1 _and Y_0 _as if they were baseline covariates, fixed from the start of follow-up. The only possible effect of exposure is then on whether we see Y_1 _or Y_0_. In other words, the exposure status X of a unit is unrelated to each *potential *outcome of the unit; exposure only determines which potential outcome we may observe. If exposure occurs (X = 1), we may observe Y_1 _but not Y_0_; if exposure doesn't occur (X = 0), we may observe Y_0 _but not Y_1_. The unobservable potential outcome (Y_0 _if X = 1, Y_1 _if X = 0) is sometimes called *counterfactual *(contrary to fact). This name is inaccurate under the strictest formulations of the model, because each potential outcome is presumed to be conceivable regardless of the exposure history, and its value may be the actual outcome regardless of the actual exposure history. For example, if a person's potential outcomes are Y_1 _= 1, Y_0 _= 1 where Y is a death indicator, the person's actual outcome is death regardless of exposure; only the unrealized *exposure *history ("exposed" when no exposure occurs; "not exposed" when exposure occurs) is counterfactual.

These somewhat odd features helped inspire vigorous attacks on the model (e.g., see the comments following Maldonado and Greenland [3]), but those were not accompanied by evidence that the model gave misleading results in any real example. On the contrary, the idea of potential outcomes fixed at baseline brings a certain transparency to and supplies logical justification for many accepted statistical procedures [27,28,31-34].

One limitation of IEEC, shared with most descriptions of potential outcomes, was that it confined its discussion to deterministic Y_1 _and Y_0 _(which were taken to be binary indicators). There is nothing essential about this simplification, however. Sequels (e.g., [2,35]) made clear that Y_1 _and Y_0 _could instead be replaced by potential *parameters *θ_1 _and θ_0 _of outcome distributions to allow for stochastic outcomes. And, as could Y_1 _and Y_0_, θ_1 _and θ_0 _could be vectors or more complex structures.

Another shortcoming of IEEC (shared by most of the literature on causation in epidemiology, especially the graphical literature) is that it did not emphasize the importance of limiting the exposure X to a potentially changeable condition, in order to make sense of the unobserved potential outcomes [36,37]. Importantly, this shortcoming was *not *shared by other articles appearing at the time [38,39]. Unfortunately, Holland's famous description thoroughly botched the model's history, misattributing it to Rubin.

#### Confounding, Collapsibility, and Exchangeability

Noncollapsibility of a measure of association over a covariate means that the measure changes upon stratification by the covariate; stated in reverse, it means we get a different measure if we collapse over (ignore) the covariate. As the above citations noted, noncollapsibility by itself could not imply confounding, since the covariate might be affected by exposure; the change would then reflect adjusting away the exposure effect or the creation of selection bias, rather than any confounding reduction. More subtly, as noted in IEEC, the change might reflect the introduction of confounding or selection bias by another, uncontrolled covariate [1,25,40-43], or it might reflect an effect of measurement error rather than confounding [44]. And of course the change might reflect only random error (the hypothesis addressed by collapsibility tests). But many authors naturally assumed that, absent these phenomena, any change must reflect removal of confounding by the covariate.

Miettinen and Cook [21] argued that this was not true in general: Some measures (those that were not differences or ratios of probabilities, such as odds ratios) could change upon adjustment even if no confounding or other bias were present. Even more striking, the converse could hold: confounding by a covariate could be present even if no change in such measures occurred upon adjustment for the covariate. Their intuition was lost on many statisticians, who continued to equate confounding with noncollapsibility. It thus seemed worthwhile to explain the source of the intuition in a more rigorous manner.

In doing so, the approach in IEEC was to take care to *not *define confounding in terms of other covariates. Rather, the strategy was to show there could be no confounding (mixing of effects) given "exchangeability" of the groups being compared (or "comparability", as Miettinen and Cook called it). The compared groups were said to be *exchangeable *with respect to an outcome measure if their outcomes would be the same whenever they were subjected to the identical exposure history. The groups were said to be only *partially *exchangeable if their outcomes would be the same when they were subjected only to certain (not necessarily all) exposure histories.

Using a table to contrast the distribution of potential outcomes in two groups (which we will here label A and B), IEEC focused on the example of a binary exposure variable X leading to binary potential-outcome variables Y_1 _and Y_0_, where the latter indicate the development of a disease. In this setting, the average outcome is the incidence proportion. The two groups would be exchangeable with respect to all-or-none exposure and average outcome if they had identical average values of both Y_1 _and Y_0 _(i.e., identical incidence when subject to the same exposure). They would thus have the same average outcome if they were both entirely exposed or if they were both entirely unexposed. They would be only partially exchangeable if the average of (say) Y_0 _was the same for both groups but the average of Y_1 _differed between the groups; in that case they would have the same average outcome when not exposed but a different average outcome when exposed.

As did Miettinen and Cook, IEEC assumed that interest focused on comparing the average outcome of an exposed (X = 1) population A to what that outcome would have been had the *same *population not been exposed (X = 0). We could observe the average of Y_1 _in A, but not the average of Y_0 _in A. We would hope to find or construct a group B that plausibly had the same average of Y_0 _as did A, and was not exposed so that we observe this average. By substituting the average of Y_0 _in B for the average of Y_0 _in A, we could now take the difference in the two observable quantities (the average of Y_1 _in A and the average of Y_0 _in B) as our measure of effect of exposure in A. But, if the Y_0 _average in B did not equal that in A, simply pretending the two were equal would create a bias in our estimate of the effect of exposure in A. That bias is what IEEC called confounding, since it mixed "baseline" differences in A and B (i.e., the difference in the Y_0 _averages in A and B) with the desired effect (the difference of the Y_1 _and Y_0 _averages in A).

As discussed subsequently to IEEC [2,3,6,24], the concepts generalize immediately to situations in which X = 1 and X = 0 represent two different exposure *distributions *or *population *(group-level) *interventions *for the target population A, to situations in which the outcome of interest is something other than an average, and to situations in which the target A is observed under *neither *pattern of interest. In the latter situations, observable substitutes must be found for the outcome of A under X = 1 and the outcome of A under X = 0, perhaps from subsets of A (as in randomized trials) or from entirely different populations. Failure of these substitutes to equal the target outcomes would normally lead to bias, which again we would call confounding.

#### Induced Confounding and Illusory Confounding

Control of intermediates (often called *mediators *in the social-science literature) is sometimes promoted as a strategy to estimate effects transmitted through pathways not involving the intermediates. Judd and Kenny ([45], p. 608-609) noted that, even in randomized trials, this strategy could be biased by failure to control for factors that affect both the intermediates and the outcome of interest. Robins [39] and Robins and Greenland [46] extended the potential-outcomes framework of IEEC to illustrate this problem, and provided no-confounding criteria for estimating direct effects; see also Pearl ([47], sec. 4.2). The problem is much more easily seen using causal diagrams, where it can be described as confounding induced by opening noncausal (biasing) paths between the exposure and the outcome due to conditioning on the intermediate [48-50].

As mentioned earlier, IEEC and the companion paper by Robins and Greenland [40] noted that confounding can be induced by control of baseline covariates. Again, diagrams better show how this confounding arises by opening noncausal paths between the exposure and the outcome [25,41,49,51]. The practical importance of the phenomenon may be limited apart from special situations [52]. Nonetheless, the examples show why one cannot be sure that confounding is being reduced as one adjusts for additional confounders and sees the estimate change: Even if the confounders satisfy the usual conditions for being a confounder and there is no other bias, it is theoretically possible that the change represents an increase in confounding.

There are other problems with examining changes in estimates to judge whether confounding is being controlled. One is that, for a common outcome, changes in odds ratios and (to a lesser extent) rate ratios may largely reflect noncollapsibility rather than confounding removal [2,24]. Another is that adjustment may induce changes in estimates solely by increasing sparse-data bias, leading to a misimpression that confounding is being reduced [53] (p.525).

### Epidemiologic Confounding, Randomization, and Ignorability

In statistics, the word "confounding" has often been used to describe related but different concepts, and does not even play a central role in many statistical discussions of causal inference (e.g., in the theory of experimental design, where "confounding" refers to an intentional design strategy). Instead, assumptions of no confounding are replaced by *randomization *or else by *ignorability *assumptions. Ignorability assumptions are sometimes called "no unmeasured confounding" assumptions, even though this usage differs from the usual epidemiologic meaning of "no confounding" (although in very large samples the two are equivalent in practical terms).

#### Randomization and Ignorability

Simple (complete) randomization of exposure means that exposure events occur independently of *every *event that precedes their occurrence. In particular, because the potential outcomes already exist at the times of exposure events, it implies that exposure events occur independently of the potential outcomes of interest. The latter independence condition is often called an *ignorability *assumption [32]; that is, ignorability is a narrowing of the randomization assumption to the specific outcome under study. For example, *weak *ignorability for a binary exposure event (X = 1 or X = 0) says exposure events occur independently of *each *potential outcome Y_1 _and Y_0_; *strong *ignorability says exposure events occur independently of the *pair *of potential outcomes (Y_1_, Y_0_). Strong ignorability implies weak ignorability, and randomization implies them both; thus any nonignorability implies lack of randomization. Each of these conditions may also be defined conditionally on some set of covariates (e.g., strong ignorability conditional on age and sex).

Ignorability of exposure events (X levels) with respect to a pair of potential outcomes Y_1_, Y_0 _says X occurs independently of Y_1 _and Y_0_. This property makes X independent of any function of Y_1 _or Y_0 _(e.g., their logs). It follows that the subpopulation defined by X = 1 and the subpopulation defined by X = 0 would be exchangeable with respect to any function or summary of the potential outcomes (e.g., the mean of Y_1_, the mean of Y_0_, or their geometric means). Thus it seemed (and still seems) to many statisticians that there should be no confounding of any measure, once we are given the ignorability condition.

Given a causal diagram for the data-generating process under study, we may ask if we can unbiasedly estimate the effect of X on Y conditional on a set of covariates Z in the diagram. It turns out that is the case if Z satisfies the *back-door criterion *of Pearl [51,54], which implies ignorability of X events with respect to (Y_1_, Y_0_) given Z. Pearl however refers to this condition as "no confounding" [25]; as discussed next, this usage diverges from our usage of "no confounding" in IEEC and at various points since [2,6,55].

#### Randomization and Ignorability versus No Confounding

There is a discrepancy between the concepts of randomization and ignorability as defined in statistics and the concepts of no confounding and exchangeability as defined in IEEC and in Robins and Morgenstern [55]. To maintain conformity with traditional usage, IEEC defined nonexchangeability and hence confounding in terms of the *actual *rather than probabilistic exposure associations with potential outcomes. Thus confounding can be present even if the exposure assignment mechanism is completely random.

To see this problem, suppose the outcome is 10-year mortality and the compared groups differ by baseline age and sex. Then it is almost certain some confounding is present, because age and sex differences will almost certainly lead to mortality differences, and those differences are not due to exposure. As noted in IEEC, it does not matter if exposure was randomized or otherwise assigned in an ignorable manner; once the assignment is made and the groups are created, any outcome differences between them will be confounded (mixed) with exposure effects [2,17,20,21,55-58].

Why do randomization and ignorability fail to capture no-confounding assumptions in epidemiology and sociology? Because randomization and ignorability refer only to the *mechanism *that generates exposure events, not to the product of that mechanism. An ignorable mechanism may by chance leave some degree of confounding in any exposure assignment it makes. As has long been recognized, most assignments made by actual ignorable mechanisms (such as simple randomizers) will have some degree of confounding in the sense of mixing of effects (e.g., [56]). Analytic adjustment for baseline covariates can remove confounding by those covariates, but will leave confounding by unadjusted covariates. This problem is sometimes called one of "unmeasured confounding" but remains a problem whether the covariate is measured or not; what matters is whether it is adjusted in a manner that removes confounding by the covariate and that does not introduce more confounding.

As the size of the randomized groups in a given trial increases, randomization does make it ever less probable that substantial confounding remains. This feature reflects a key statistical advantage flowing from successful randomization: The confidence limits capture uncertainty about the confounding left by randomization [33,55]. In this sense, randomization-based confidence limits account for concerns about residual baseline confounding by unmeasured factors in randomized trials, although post-randomization events (such as censoring) may reintroduce these concerns. Note that other statistical aspects of the trial (e.g., number of participants, power) do not enter into these considerations except through confidence limits, so that a trial that is "large" and "powerful" may nonetheless be poorly informative because it produced a wide confidence interval.

#### Adjustment for Baseline Covariates under an Ignorable Mechanism

As noted above, there have been many intuitive and mathematical arguments as to why adjustment for baseline covariates can be important, even with an ignorable exposure-assignment mechanism such as randomization (e.g., [17,20,21,55,57-59]). This importance is reflected by the fact that in propensity-score (exposure-probability) adjustment for confounding, adjustment by the fitted score provides better frequency properties than the true score [32,60]. For example, under simple 50% randomization to exposure, the true propensity score is constant across all individuals (it is 0.5, the chance of being assigned to exposure); its use thus produces no adjustment at all, since everyone will end up in the same score stratum. In contrast, a fitted score will adjust for the covariates it includes, although the extent of that adjustment may depend heavily on the form of the propensity model.

In sum, randomization (or more generally, ignorability) does **not **impose "no confounding" in the common-sense use of the term. Rather, it provides the following related properties [1,33,55,58]:

1) *Unconditional expected *confounding of zero: This is a pre-allocation expectation, corresponding to no asymptotic bias over the randomization distribution; but once allocation occurs, it becomes secondary to properties of the allocation.

2) A randomization-based ("objective") derivation of a prior for *residual *confounding *after conditioning on all measured confounders*. This prior applies after allocation as well as before, and becomes more narrowly centered around zero as the sample size increases. This is a key post-allocation benefit of randomization.

As emphasized by R.A. Fisher [61], it also provides a randomization-based distribution for conducting frequentist inference on effects [62]; property (2) can be viewed as a Bayesian version of this property [58].

#### Exchangeability in IEEC and Subjective Probability Theory

Property (2) above was the basis for IEEC tying the epidemiologic concept of confounding to the subjective-Bayesian concept of exchangeability. In probability, random variables are said to be *exchangeable *(under a given joint distribution) if they can be interchanged (permuted) in any statement without altering the probability of the statement [63]. For example, if we consider U and V exchangeable with joint probability distribution Pr(U, V), the probabilities Pr(U<V) and Pr(V<U) derived from Pr(U, V) must be equal. Exchangeable random variables are in essence indistinguishable with regard to our bets about their values (whether absolutely or relative to one another). Note that exchangeability is a property of the joint distribution of the variables. In subjective probability systems, different observers may have different views on whether the variables are exchangeable because they may assign different distributions to variables.

In IEEC, the random variables at issue are the distributions of potential outcomes in the compared groups A and B, and the discussion focused on a binary deterministic outcome. To describe the situation more generally (as in [2]) for a binary exposure X, let U_1A _and U_0A _be some summary of the outcomes in group A when everyone in A is exposed (X = 1) and when everyone in A is unexposed (X = 0); similarly for group B, let U_1B _and U_0B _be the summaries when everyone in group B is exposed and when everyone is unexposed. X might be an individual exposure (e.g., carbohydrate consumption) or a group-level exposure (e.g., legislation, insurance). Further, assume that what happens in each group has no effect on what happens in the other, but that U_0A _and U_0B _are exchangeable given U_1A_. Then in drawing inferences about the effect of exposure (of X = 1 vs. X = 0) on A, say U_1A_-U_0A_, we could substitute U_0B _for U_0A_.

The Bayesian exchangeability connection provides one explanation (beyond common-sense and abstruse ancillarity arguments) for why we might declare confounding to be present in a study even though the mechanism that assigned the exposures was ignorable. Because Bayesians condition on all the observed data, once we observe an association of a baseline risk factor with exposure, exchangeability is lost for us: Upon observing an association, we assign different distributions to U_0A _and U_0B_, reflective of the information suggesting a baseline difference.

Note well that this version of exchangeability is a property of our information about the groups and the variables, hence is a subjective (observer-relative) property, not an absolute (objective) property of either. In this view, evaluation of confounding is equally subjective.

### Our Research on Confounding Since IEEC

Since IEEC we have separately pursued quite different paths to deal with problems that involve confounding by many covariates. Our divergence stems primarily from differences in the applied settings and target problems that have been our focus over the decades since IEEC. Nonetheless, it has at times raised a few interesting philosophical issues, especially when dealing with many exposures or confounders.

#### Multiple Exposures as Multiple Confounders

Consider first a study involving single measurements of multiple exposures, as is common in case-control studies of occupation and lifestyle. In these settings each exposure may be a potential confounder for every other exposure, so it is natural to consider an outcome-regression model containing all exposures. In conventional theory, this sort of model could provide an estimate for each exposure "adjusted" for all the rest. Unfortunately, it often happens that the number of subjects available may appear large but is not large enough to produce approximately unbiased estimates from the usual maximum-likelihood (whether unconditional, conditional, or partial) or estimating-equation methods [64]. These problems arise because the number of subjects needed to get approximate unbiasedness grows exponentially with the number of covariates in the model. In some applications the usual estimators fail completely due to collinearity [65-67].

One way to address these problems is to accept that (in observational studies) some bias is unavoidable, and may even be tolerable in exchange for improved accuracy on average over all the exposures being considered. The usual methods then become unacceptable because they can incur huge bias without a compensating accuracy benefit. Methods that pursue over-all accuracy improvement include the vast array of multilevel and hierarchical techniques developed under the headings of ridge regression, shrinkage (Stein) estimation, penalized estimation, and empirical and semi-Bayes estimation (see [68] for an elementary overview). These methods have also been proposed as replacements for conventional variable-selection procedures in order to avoid the distortions produced by selection [53,69]. Finally, the methods have been extended to study the impact of uncontrolled confounding and other biases in observational research [70-73].

#### Time-Varying Exposures

At the time of IEEC, one of us (Robins) was developing methods estimating effects of time-varying treatments from longitudinal data. Early accessible introductions in this work include Robins [39,62] and Robins et al. [74]; see also Robins et al. [75]. Estimating effects of potentially complex treatment histories created extreme difficulties for likelihood-based methods (which were standard at the time). These difficulties led to a frequentist approach with confounder adjustment based on regression of treatment probabilities on measured confounders (as in propensity-score methods) followed by use of this fitted treatment model for adjustment. Fitting is accomplished by semi-parametric methods such as g-estimation and inverse-probability-of-treatment (IPT) weighting. These methods were further extended in many directions, including control of confounding due to treatment noncompliance [76], estimation of direct effects [77-79], and to the study of uncontrolled confounding [80-82].

In the course of this IPT development, it was shown that familiar likelihood-based methods (including maximum-likelihood and Bayesian outcome regression) could exhibit poor large-sample frequency properties if many covariates needed adjustment and no use was made of treatment probabilities in fitting [83,84]. These observations have led to recommendations for doubly robust procedures that fit outcome regressions using IPT weighting [85,86]. This approach resembles recommendations that outcome regressions be conducted after propensity-score matching has removed gross confounder imbalances between compared groups [32]. The shared intuition is that if one of the regression models (for treatment or outcome) is mis-specified, the resulting adjustment failures will be compensated for by the adjustment induced by the other model.

A topic worthy of further research that we have long recognized but not pursued is how to blend ideas from our divergent methods and applications. Some applications of shrinkage-like methods, such as boosting to predict treatment, appear useful for taming problems due to unstable weights [87]. One logical next step might be to apply shrinkage methods to the IPT-weighted outcome regression as well as to the weight regression. We are not aware of studies of such "doubly robust double shrinkage."

## Conclusion

Some colleagues have said that they think IEEC is among the most important methods papers from its era, and it is often, though not always, cited in reviews of epidemiologic confounding, e.g., in Nurminen [7] and Pearl [25] but not in Vandenbroucke [5]. Regardless of its historical status, IEEC is a snapshot of a topic in transition. Using a standard causal model familiar to experimental statisticians since the 1930s, IEEC provided a formal definition of confounding and logical justifications for traditional intuitions about confounding graphed in earlier literature [14,16-18]. It also provided logical justifications for less intuitive distinctions such as that between confounding and collapsibility, and that between nonconfounding and randomization or ignorability.

The terminology in IEEC could have been clearer; for example, it talked of "causal confounding" without ever explaining what noncausal confounding would be. The phrase "causal confounding" was a nod to the fact that some researchers also talked of "selection confounding," in which bias arose because a covariate related to exposure affected selection rather than disease; today we would simply call this phenomenon selection bias. There also could have been a clearer connection made to the topic of standardization and its relation to the target population for inference. Nonetheless, the framework in IEEC served as a basis for many of the more general and more extensive treatments that followed [2,3,33,35,46]. We hope that its sequels rectified the shortcomings of IEEC, and that modern readers come away from it feeling equipped to move on to subsequent literature without too much difficulty.

## Competing interests

The authors declare that they have no competing interests.

## Authors' contributions

SG wrote and revised the manuscript. JMR suggested revisions. Both authors read and approved the final manuscript.
